# HPV and Penile Cancer: Epidemiology, Risk Factors, and Clinical Insights

**DOI:** 10.3390/pathogens13090809

**Published:** 2024-09-18

**Authors:** Gowtam Mannam, Justin W. Miller, Jeffrey S. Johnson, Keerthi Gullapalli, Adnan Fazili, Philippe E. Spiess, Jad Chahoud

**Affiliations:** 1 USF Health Morsani College of Medicine, Tampa, FL 33602, USA; gmannam@usf.edu (G.M.); justinmiller1@usf.edu (J.W.M.); 2 Department of Genitourinary Oncology, H. Lee Moffitt Cancer Center and Research Institute, Tampa, FL 33612, USA; jeff.johnson@moffitt.org (J.S.J.); keerthi.gullapalli@moffitt.org (K.G.); adnan.fazili@moffitt.org (A.F.); philippe.spiess@moffitt.org (P.E.S.)

**Keywords:** human papillomavirus, HPV, penile cancer, penile tumor, PSCC, immunotherapy, tumor immune microenvironment

## Abstract

Penile cancer (PC) is a rare malignancy predominantly of squamous cell origin. Approximately 40% of penile tumors are associated with human papillomavirus (HPV) infection. Diagnosing PC remains challenging due to its rarity and variety of clinical presentations. Furthermore, the impact of HPV on the tumor immune microenvironment complicates clinical management, although recent advancements in immune checkpoint inhibitors (ICIs) have shown some efficacy in treating HPV-associated PC. Ongoing research efforts aim to develop oncologic treatments that target HPV-induced cellular modifications. Additionally, novel therapeutic vaccines and adoptive T-cell therapies targeting HPV oncoproteins represent emerging treatment modalities. Our review highlights the complex interplay between HPV and penile carcinogenesis, emphasizing its epidemiology, etiology, clinicopathological characteristics, and potential therapeutic implications.

## 1. Introduction

Penile cancer (PC) is a rare malignancy of penile tissues. The estimated age-standardized incidence rate (ASR) of PC in 2020 was 0.8 per 100,000 globally, with higher rates in South America, Southern Africa, and South Asia, where incidence can account for up to 10% of malignancies [1,2]. In the United States, the incidence of PC is 0.38 per 100,000 with a mean age of 67 [2,3]. Most penile cancers are of squamous cell origin with frequent association with the human papillomavirus (HPV) [4]. Consequently, penile squamous cell carcinoma (PSCC) is generally classified as being either HPV-associated or HPV-independent. Other risk factors include phimosis, tobacco use, poor hygiene, sexual promiscuity, and a low socioeconomic status [5]. HPV-associated penile cancers account for 38.5% of cases, with the HPV 16 serotype being the most common [6]. Despite tumor size, invasion, nodal involvement, and the presence of metastases being the primary prognostic factors, knowledge of HPV pathogenesis provides a strong target for PC treatment [7,8]. In this review, we aim to provide an overview of HPV-associated penile cancer to summarize its clinicopathological characteristics and emerging clinical applications targeting HPV.

## 2. Morphology, Epidemiology, and Risk Factors of Penile Cancer

Penile cancer is classified into histological subtypes in accordance with its morphological features and association with HPV. Histological subtypes are divided primarily into two categories: HPV-associated and HPV-independent subtypes [9]. Basaloid and warty penile squamous cell carcinomas have a high prevalence of HPV and are considered to be associated with HPV. Usual, papillary, and verrucous carcinomas have a low prevalence of HPV and are considered HPV-independent [10].

HPV-associated and HPV-independent subtypes can primarily be distinguished through morphological differences in H&E staining. The basaloid subtype is distinguished by the presence of basal immature cells with a high nuclear-to-cytoplasm ratio or scant cytoplasm [11]. The warty or condylomatous subtype is grossly distinguished with a cauliflower appearance and koilocytic atypia [11]. In complex cases, immunohistochemistry (IHC) is used to identify HPV in lesions. The p16INK4a protein is a cyclin-dependent kinase inhibitor that is overexpressed in HPV-induced neoplasia due to the inactivation of Rb by the HPV E7 protein [12]. Immunohistochemistry using P16INK4a has 100% specificity and positive predictive value, which makes it a valuable tool in HPV detection [13].

HPV-associated subtypes are considered high-risk due to their greater potential for lymph node involvement and metastasis [14]. High-risk subtypes have a 28-fold increased relative risk of inguinal lymph node involvement, which makes histological grading important to identify HPV involvement and prognosis [15]. Patients with basaloid, warty, mixed warty–basaloid, clear cell, and other poorly differentiated lesions will be more likely to benefit from prophylactic inguinal lymph node dissection (ILND) [14].

The prevalence and incidence of penile cancer have varying trends globally. PC represents 1% of all malignancies in US men and 0.1% of all deaths related to malignancies [16]. Historically, incidence rates in the US have been decreasing since the 1970s and have stabilized to 0.38 per 100,000 from 2000 to 2018 [2,17]. The 5-year relative survival rate has also remained consistent in the US from 2000 to 2014, ranging from 65.67% to 67.70% [2]. Racial differences in incidence were also observed from 1995 to 2003, where Hispanic men (6.58 per 1,000,000) had the greatest incidence and Asian Pacific Islanders (2.40 per 1,000,000) had the lowest incidence [18]. Patients may hesitate and delay seeking medical attention due to embarrassment, fear, or denial, which reduces their ability to retain function after treatment and lowers their chances of survival [19].

In other countries, penile cancer presents a more significant burden. In India, PC has an incidence rate of up to 3.32 per 100,000, while Northeast Brazil has the highest incidence globally at an ASR of 6.1 per 100,000 [2,20]. A regional analysis of PC incidence in Brazil shows high variance in relation to socioeconomic status and literacy. In Northeast Brazil, penile cancer is responsible for 5.7% of neoplasms in men, differing greatly from the 1.2% in South Brazil [21]. This disproportionate distribution of PC along with its rarity presents challenges to studying the disease as areas with the highest prevalence lack recruitment into trials [22].

A meta-analysis accumulating data from 1995 to 2022 found that the global pooled prevalence of HPV in men was 31%, with 21% being high-risk HPV [23]. The prevalence varies between developing (42.2%) and developed (22.6%) countries; however, there is no indication that HPV-associated penile cancer is more prevalent in developing countries [24]. In PC, there is a 50.8% prevalence of HPV DNA [25]. One study reported that HPV-associated penile cancers showed improved disease-specific 5-year survival rates [7]. This frequent presence of HPV provides a mode of prevention and treatment against penile cancer.

Phimosis, the inability to retract the foreskin covering the glans of the penis, is a notable risk factor for the development of penile cancer. Phimosis was observed in 35.2% of penile cancer cases in comparison to 7.6% in the controls [26]. Phimosis occurs in uncircumcised and improperly circumcised individuals. In a systematic review of 248 cases and 2959 controls, circumcision in childhood or adolescence had a protective effect by reducing the incidence of PC with an odds ratio (OR) of 0.33 [27]. Phimosis is conducive to the accumulation of smegma, which is the secretion of the sebaceous gland consisting of shed skin cells, skin oils, and moisture that deposits under the foreskin [28]. Therefore, phimosis exacerbates poor hygiene by maintaining excess smegma, leading to chronic irritation and a tumorigenic environment [19].

Tobacco use, including smoking, has been determined as an additional risk factor for penile cancer. Smoking has a dose–response relationship, where smokers consuming more than 10 cigarettes a day had a significantly higher risk for PC compared to those consuming 1–10 cigarettes a day [29]. Tobacco carcinogens collect in the smegma secretions of the penis, creating a microenvironment for carcinogens to induce increased DNA damage per mutagenic event [30]. Furthermore, current smokers have an increased risk for HPV (OR = 1.19) and oncogenic HPV (OR = 1.24), eliciting a compounded effect on the risk of penile cancer by simultaneously elevating the incidence of HPV [31]. HPV prevalence is further enhanced by the number of sexual partners, another prominent PC risk factor [32].

## 3. Genitourinary Manifestations of Human Papillomavirus in Males

In a 2014 study, the prevalence of HPV in men aged 14–59 in the US was 42.2%, with the prevalence of HR-HPV being 23.4% [33]. Most HPV infections in men are asymptomatic or subclinical, with the prevalence of HPV in asymptomatic men ranging from 1.3 to 72.9% [34]. The virus remains latent in genital tissue, and only HPV DNA is detected with potentially microscopic histological changes. HPV was more often detected in samples from the shaft of the penis (49.9%), glans (35.8%), and scrotum (34.2%), but less likely detected in the perianal area (20.0%), anal canal (17.6%), urethra (10.1%), and semen (5.3%) [35].

Genital warts are the most common clinical presentation caused by HPV infection in men. Although genital warts are primarily benign lesions of which 90% are associated with LR-HPV types 6 and 11, they can limit patients psychosocially and are highly infectious [36]. Approximately 65% of individuals whose sexual partners have genital warts will develop them as well [35]. These lesions present as papillary, coronal, or cauliflower-like masses [37].

HPV positivity in semen has shown a significant association with the risk of infertility in men (OR: 2.93) [38]. Sperm progressive motility was found to be reduced in patients infected with seminal HPV [39]. Reduced motility is partially explained by the adhesion of HPV using capsid protein L1 to bind to sperm glycosaminoglycan syndecan-1 [40].

HPV prevalence in the anal canals of heterosexual men was 12.0%, 7.0% of which is the oncogenic type dominated by HR-HPV 16. In a meta-analysis pooling 18,646 men and women with and without anal cancer, HR-HPV type 16 was determined to be carcinogenic with a dose–response relationship, indicating that increased HPV 16 positivity correlates with an increased severity of lesions [41].

## 4. Pathogenesis of Human Papillomavirus-Related Penile Cancer

The pathophysiology of HPV-associated penile cancer is better understood than that of HPV-independent penile cancer. HR-HPV 16 binds to Heparan Sulfate Proteoglycans (HSPGs) and undergoes conformation changes to initiate early virus–host cell interactions [42]. The virus also interacts with α6-integrins to evoke intracellular signaling cascades that allow for early viral genome amplification [42]. With constant exposure to HPV, the penile tissue cells will integrate the HPV genome into the host cell genome, leading to an overexpression of HPV oncoproteins E6 and E7 [43].

The E6 and E7 oncoproteins are crucial for HPV-induced carcinogenesis. E6 interferes with tumor suppressor protein p53 and induces an increase in its ubiquitin-dependent proteasomal degradation, leading to cell cycle destabilization [44]. Similarly, E7 interferes with tumor suppressor protein Rb and induces its ubiquitylation, leading to proteasomal degradation. This frees transcription factor E2F from its Rb-E2F complex to translocate to the nucleus and promote a malignant phenotype [44]. The unregulated multiplication of HPV-infected penile cells yields an overexpression of p16INK4a, which serves as a prognostic marker in HPV-associated cancer [45]. Furthermore, E7 downregulates tumor suppressor proteins p21 and p27 to result in uncontrolled activity of Cyclin A and E to promote cell cycle progression [46].

HPV-induced DNA hypermethylation of the host cell genome is another method the virus uses to selectively repress tumor suppressor genes in favor of oncogenes [47]. An analysis of these epigenetic changes showed that they can serve as biomarkers for HPV-associated penile cancers. One genome-wide analysis revealed that over 71% of methylation variable positions (MVPs) on tissue cell genomes were hypermethylated compared to normal tissue [48]. MVPs were grouped into functionally important clusters named Differentiated Methylated Regions (DMRs). A further analysis determined 1255 significantly different DMRs in malignant tissue compared to normal tissue [48]. Specific tumor suppressor genes (TSGs) that are hypermethylated alterations include CDO1, AR1, and WT1, which may lead to dysregulation and carcinogenesis [48]. Targeting the epigenetic modifications that propagate malignancy may provide new therapeutic interventions to improve patient outcomes.

## 5. Clinicopathological Characteristics of Penile Cancers

Penile cancer can manifest through different lesions located primarily on the foreskin, inner foreskin mucosa, and glans penis with potential invasion into the corpus spongiosum and corpora cavernosa [49]. Lesions can present as flat or endophytic, ulcerated, indurated, as papules or warts, and as erythroplasia. Exophytic lesions are common in advanced cases; however, PC has diverse clinical presentations beginning with precursor penile intraepithelial neoplasia (PeIN), which can be limited to subtle changes [43,50].

The major difference in manifestation between HPV-associated and HPV-independent PC in histological subtype is the appearance of lesions. HPV infection manifests as PeIN with basaloid, warty, or warty–basaloid features, indicating a high-risk subtype associated with HPV [7]. These precursor lesions will also exhibit upregulated p16INK4a if induced by HPV and may worsen to penile squamous cell carcinoma.

## 6. Staging and Grading of Penile Cancers

Tumor staging and grading assist in defining the invasion and aggressiveness of all tumor types. Penile tumors are staged in accordance with the AJCC Cancer Staging Manual, which classifies tumor size and invasion extent (T), lymph node involvement (N), and distant metastases (M) [51]. Currently, HPV status does not play a role in staging penile cancer. To properly stage, and thus treat, PSCC, a physical exam is essential. Clinical staging is carried out by a manual evaluation of the tumor size and local nodal metastasis. For patients with no clinically evident regional lymph nodes, high-risk lesions (T1b or greater) should be offered surgical lymph node staging. In the case of clinically evident lymph nodes, an image-guided biopsy should be offered to confirm nodal metastasis prior to the initiation of treatment. For local staging and tumors invading the corpora cavernosa, magnetic resonance imaging (MRI) provides a precise estimate of the tumor infiltration depth that coincides well with histopathology findings [52]. In patients with nodal disease, F-fluoro-2-deoxy-D-glycose positron emission computed tomography (FDG PET/CT) of the chest and abdomen should be carried out before the initiation of treatment to evaluate the presence of distant disease [53]. In clinically node negative patients, however, PET/CT alone is typically not sensitive enough and should be carried out in combination with a sentinel lymph node biopsy to reduce the likelihood of false negative results [54].

While HPV status currently does not play a role in the staging of penile cancer, in 2022, the World Health Organization (WHO) recommended the classification of PeIN into HPV-associated and HPV-independent subtypes [55]. Subsequently, the European Association of Urology-American Society of Clinical Oncology Collaborative (EAU-ASCO) guideline update from 2023 states that it is mandatory to report the status of p16 in the pathology report [53]. HPV has a major impact on disease management and prognosis in other cancers dependent on HPV (for example, head and neck squamous cell carcinoma). However, in PSCC, there is insufficient data to guide treatment [56]. This change in the pathologic reporting of penile cancer is an effort to remedy the lack of data in the hopes of ultimately elucidating the impact of HPV on prognosis and, subsequently, disease staging and management.

## 7. Pathogenesis of Human Papillomavirus and Prognostic Value of p16 Protein and Other Biomarkers in Penile Cancers

HPV is a non-enveloped, circular, double-stranded DNA virus that enters the epithelium through the skin and mucosa to eventually infect the proliferating basal layer of keratinocytes [57]. The transmission of HPV primarily occurs through sexual intercourse, with each sexual encounter presenting a 40% probability of HPV transfer [58]. Non-sexual transmission is possible via skin-to-skin or skin-to-mucosa contact [59]. Following transmission, the HPV genome typically persists as extrachromosomal DNA in host cells. Over time, in some cases, the HPV genome integrates into host cells to influence the host cell genome, manipulate cellular functions, and contribute to a tumorigenic transformation [60]. However, integration is associated primarily with high-risk serotypes and is not necessary for carcinogenesis, as HPV-associated cancers may occur when the genome remains episomal [61].

The HPV genome is 8 kilobases in size, divided into an early region, a late region, and a non-coding long-controlling region [62]. The early region encodes proteins E1–E7, which are crucial for viral replication and pathogenesis [63]. The late region encodes proteins L1 and L2, the major capsid and minor capsid proteins, respectively. L1 is responsible for forming the capsid structure to encapsulate the genome and to provide protection during infection [64]. L2 mediates intracellular interactions to promote viral internalization and conduct viral trafficking [65]. After the initial infection, the productive phase begins viral replication, amplifying the virus to 10 to 200 copies per cell and upregulating the expression of E1 and E2 [66]. E1 and E2 control genome replication and transcription. In early infection, E2 transcriptionally represses E6 and E7 [67]. E4 maintains the genome during the latent phase, and E5 inhibits the immune response, leading to HPV remaining dormant within infected cells [68,69]. E6 and E7 are oncoproteins that inhibit the regulating function of cell cycle proteins p53 and Retinoblastoma protein (Rb), leading to uncontrolled growth [70].

There are more than 200 known types of HPV, which are grouped into low-risk (LR) and high-risk (HR) based on DNA sequencing and association with carcinogenesis [71]. The most common LR-HPV serotypes are 6 and 11, whereas the most common HR-HPV serotypes are 16 and 18, which, together, have led to the development of the HPV quadrivalent vaccine providing immunity against those four serotypes [72]. HPV vaccination is primarily used to prevent cervical cancer but has the potential to alleviate HPV-induced carcinogenesis in other cancers as well. Both LR and HR-HPV have been associated with penile cancer; however, HR-HPV has a greater prevalence. HPV 16 is the most prevalent serotype, accounting for 46% to 62.5% of HPV-associated penile cancers [73,74]. Gardasil 9 (nonavalent), Gardasil (quadrivalent), and Ceravix (bivalent) are all protective against HR-HPV serotypes 16 and 18, which are the most oncogenic in penile cancer. Therefore, the administration of the HPV vaccine in males can reduce the onset of penile cancer [75]. The Advisory Committee on Immunizations Practices recommends all boys between ages 11 and 12 to receive the HPV vaccine with a catch-up vaccine at age 21 [76].

The body of research assessing the prognostic significance of p16 in penile cancer is growing. Patients that are both HPV- and p16-positive have shown improved cancer-specific outcomes; however, more data are needed to confirm the extent to which p16 status influences prognoses. In non-penile cancer HPV-associated SCC cancer types, such as head and neck cancer, p16 positivity is known to be an independent prognostic factor [77]. A single European network study involving 137 patients evaluated PeIN disease-free survival (DFS) in p16 positive versus negative patients receiving imiquimod, 5-fluorouracil, and/or surgery and found that patients with p16 positivity had longer DFS regardless of treatment modality [78]. In a single-center retrospective study involving 109 patients diagnosed with PSCC and treated with surgical resection, HPV p16 positivity predicted a 2-year locoregional control benefit over HPV p16 negativity when subsequently treated with adjuvant chemoradiotherapy [79]. Retrospective studies have found that HPV-associated penile cancers exhibit greater radiosensitivity and result in more favorable outcomes compared to HPV-independent cancers [7,77,80,81]. Perioperative radiation was assessed in a retrospective international multicenter study involving 507 patients. In this study, patients with HPV-associated penile cancer who received perioperative radiation (primarily adjuvant) had significantly longer median overall survival rates [82]. Generally, the 5-year disease-specific survival was found to be superior in high-risk HPV invasive penile cancer as opposed to HPV-independent penile cancer in a retrospective study involving 212 patients [83]. Furthermore, a histologic analysis of tumor samples found that p16 positivity was associated with a longer median cancer-specific survival and a significantly higher probability of 5-year cancer-specific survival [84]. Two retrospective reviews, with 123 and 43 patients, respectively, indicated that p16 positivity was correlated with a statistically significant improvement in overall survival [85,86]. A recent systematic review confirmed the prognostic significance of p16 and HPV, but notes that a larger, updated meta-analysis is needed to better understand the prognostic role of these biomarkers and how they can be utilized to improve treatment [87]. A meta-analysis measuring HPV and p16 positivity as markers of prognosis found that patients positive for p16 had an overall survival benefit in a subset of the studies analyzed; however, HPV positivity did not share the same association. This discrepancy in overall survival highlights a possible discordance between the two biomarkers and suggests that p16 may be more prognostic for penile cancer [88].

Additional biomarkers have been identified which may aid in the development of more specific and effective therapeutics for penile cancer. Caveolin-1 (CAV-1), a membrane protein which regulates carcinogenic processes ranging from cell transformation to metastasis, was shown to be amplified on solid tumors, especially epithelial malignant cells [89]. Forty-three patients with PSCC were evaluated for CAV-1 using immunohistochemistry (IHC), which demonstrated CAV-1 overexpression in the epithelial cells and a reduction in stromal cells. This widened epithelial–stromal CAV-1 gap in penile cancer was associated with a worse prognosis [90]. Furthermore, the epithelial–stromal CAV-1 gap was more pronounced in p16-negative patients, indicating its prognostic value in non-HPV-associated PC. In HPV-associated PC, Nectin-4 expression is significantly greater than non-cancerous tissue and demonstrates greater intensity in high-risk HPV [91]. In one case, a patient with metastatic penile cancer refractory to chemotherapy was treated with Enfortumab Vedotin (EV), an anti-Nectin-4 antibody conjugated with microtubule-disrupting agent monomethyl auristatin E (MMAE), which has been approved for advanced urothelial cancer [92]. This patient had significant symptomatic improvement with less toxicity in comparison to a prior chemotherapeutic treatment, which warrants the development of larger clinical trials of EV and other Nectin-4 therapeutics in PC. MicroRNAs (miRNAs) have also been investigated to identify unique expression profiles that might confer prognostic potential in PC. In 24 cases evaluated, miR-1, miR-101, and miR-204 were under-expressed, while miR-223-3p and miR-107 were overexpressed [93]. These trends were associated with greater lymphovascular invasion and a worse prognosis. Other biomarkers, such as the albumin-to-alkaline phosphatase ratio, are under exploration for their novel prognostic potential [94]. A further exploration of biomarkers is needed to improve patient outcomes, especially in subsets of patients where conventional treatment regimens fail.

## 8. The Influence of Human Papillomavirus on the Tumor Immune Microenvironment

Prior research in cervical, anal, and oropharyngeal cancers has helped elucidate the influence of HPV on the tumor immune microenvironment (TIME) [95,96,97]. An analysis of peripheral blood mononuclear cells (PBMCs) revealed that the HPV-induced overexpression of E6 and E7 proteins may inhibit IFN-α production in natural killer (NK) cells through the interruption of IL-18/IL-18R binding [98,99]. The disruption of IL-18 binding may generate additional immunomodulatory effects, including decreased stimulation of IL-2 and chemokines and reduced cytotoxicity of NK and T-cells [99]. Additionally, penile cancer immune cell markers have indicated that increases in lymph node involvement are associated with greater proportions of tumorigenic M2 macrophages and decreases in anti-tumor lymphocytes, suggesting a diminished immune response in higher-stage penile cancers [100]. The cumulative effect of these modifications to the tumor immune microenvironment likely promotes immune exhaustion and the evasion of HPV-associated cancer cells, especially in poorly differentiated tumors.

Paradoxically, the immunophenotyping of penile cancer has shown increased immune cell infiltration in HPV-associated PSCC [101]. In support of these findings, studies have correlated HPV positivity with greater amounts of tumor-infiltrating lymphocytes (TILs), higher levels of immune activation, and increased expression of perforin and granzymes [100]. Furthermore, an analysis of T-cell subtypes in HPV-positive head and neck squamous cell carcinoma (HNSCC) revealed a significantly higher M1/M2 macrophage ratio, suggesting a pro-inflammatory tumor microenvironment [102]. The improved efficacy of immune cell infiltration has been theorized to result from an increased recruitment of immune effector cells in response to the immunogenicity of HPV-associated PC [103].

Despite harboring a complex TIME, HPV-associated cancers appear to have improved prognoses following treatment with immune checkpoint inhibitors (ICIs) in comparison to their HPV-independent counterparts. In a study of advanced PSCC treated with first-line anti-PD-1 combination therapy, HPV-associated tumors showed a significantly improved overall response rate (ORR) compared to HPV-independent tumors [104]. These findings are bolstered by studies in head and neck cancers, which have shown that treating HPV-positive HNSCC with ICIs resulted in improved ORR, overall survival (OS), and risk ratios (RR) [105]. Furthermore, a meta-analysis of both PSCC and HNSCC treated with ICIs showed an improved ORR for patients with HPV [106]. A heightened response to immunotherapy in HPV-positive cancers is likely caused by the immune reaction to viral antigens, the improved infiltration of immune cells, and a more inflamed tumor microenvironment that can be better targeted by ICIs; however, specific mechanisms have yet to be described.

## 9. Current and Future Therapies for Penile Cancer and Human Papillomavirus-Associated Penile Cancer

Currently, there are limited data to guide the use of cancer-directed therapy in the treatment of HPV-associated penile cancer. As such, front-line therapy is conducted independent of HPV/p16 status. We will briefly describe standard approaches to local, locally advanced, regional, and metastatic/relapsed disease. For early disease, the treatment is local. Surgical treatment allows for complete pathologic staging; however, other regional therapies such as 5-fluorouracil imiquimod or ablation are important non-invasive alternatives in PeIN [53]. The emphasis of treatment in early-stage disease is organ preservation. In locally advanced disease without nodal spread, glansectomy is an important therapeutic approach. Additionally, radiotherapy has also been used for locally invasive disease with good results [107,108]. Once disease has spread to the corpus cavernosum, partial or total penectomy is recommended [109]. For tumors that are unresectable, neoadjuvant chemotherapy (NAC) may be an option. NAC was reviewed in a meta-analysis from 2022 evaluating 14 studies. They found weak evidence that patients who had an objective response to NAC obtained better survival outcomes in the forms of 2- and 5-year overall survival rates; however, those patients also experienced significant toxicity. Additionally, the meta-analysis showed that better overall survival outcomes were achieved after surgery for patients who demonstrated stable disease after NAC [110]. NAC generally consists of triplet therapy in the form of paclitaxel, ifosfamide, and cisplatin (TIP) modeled after a phase II clinical trial of 30 patients with stage III or IV disease (N2 or N3) which was conducted using this regimen. The study found that 50% of patients had a favorable response, and 22 of 30 patients went on to have surgery [111]. In advanced lymph node disease, such as bulky inguinal lymphadenopathy or pelvic lymphadenopathy, NAC is further supported by a meta-analysis from 2020. This analysis evaluated 10 studies, ultimately suggesting that for bulky lymph node disease, NAC should be considered prior to ILND [112]. In regional lymphatic disease, one single-center prospective study showed that adjuvant radiation improved overall survival when compared to chemotherapy in 45 patients who received lymph node dissection [113]. In metastatic disease, chemotherapy is the primary treatment modality, with TIP being the preferred regimen, and there is an emphasis on quality of life with the intent of achieving palliation [109].

While HPV/p16 status currently does not play a role in front-line treatment, its impacts on pathogenesis and prognosis illustrate an interesting opportunity for targeted treatment in the future. There are multiple clinical trials underway that aim to investigate novel treatment regimens for HPV-associated PC and other HPV-associated cancer types (Table 1).

Therapeutic HPV vaccines are being developed to treat those with active HPV-associated penile cancer. Oncoproteins E6 and E7 are vital for the oncogenic ability of HPV. Therapeutic HPV vaccines leverage this dependence by aiming to induce an immune response against the oncoproteins by stimulated CD8+ T-cells to recognize and kill HPV-positive cells [114,115]. These oncoproteins can also be targeted using adoptive T-cell therapy. This process harvests autologous tumor infiltrating lymphocytes (TILs) and expands them to be reinfused into the patient [116]. Development is currently underway to generate a T-cell receptor (TCR) that can bind to oncoproteins E6 and E7, allowing isolated TILs to target HPV-infected tumor cells for more directed delivery [117]. Both developmental procedures display the biochemical advantage that HPV-related markers present in penile carcinogenesis. Exploratory therapeutic targets involved in tumorigenesis and progression, such as nectin-4 and heat shock proteins (HSPs), may provide additional treatment modalities in the coming years [118]. These novel therapies combined with an improved understanding of the tumor immune microenvironment in HPV-associated penile cancer will help provide patients with a more robust and personalized treatment protocol.

## 10. Conclusions

HPV-associated penile cancer presents unique challenges and opportunities in its diagnosis and treatment. Understanding the multifaceted role of HPV in penile carcinogenesis and the effects of the virus on the tumor immune microenvironment are crucial for developing novel prevention and treatment strategies. Continued research into HPV pathogenesis and immune interactions will aid in the development of more personalized approaches in managing this malignancy.

## Figures and Tables

**Table 1:** Summary of ongoing, forthcoming, and recently completed clinical trials for HPV-targeted cancer therapies.

NCT *	Treatment(s)	Trial Phase	Patient Population	Trial Status
NCT05639972	Conditioning regimen (cyclophosphamide + fludarabine), E7 TCR-T cells, high-dose aldesleukin	I/II	Locoregionally advanced HPV-associated cancers	Not yet recruiting
NCT05976828	IBRX-042 (E6, E7 oncolytic virus vaccine)	I	HPV- or p16-positive carcinoma post-first-line therapy	Not yet recruiting
NCT05826275	TGN-S11 (E5 oncogene inhibitor), pembrolizumab	I	Relapsed, resistant, or metastatic HPV-associated SCC	Recruiting
NCT05686226	Conditioning regimen (cyclophosphamide + fludarabine)	II	Metastatic or refractory/ recurrent HPV-16+ cancer	Recruiting
NCT03260023	TG4001, Avelumab	I/II	Metastatic or refractory/ recurrent HPV-16+ cancer	Active, not recruiting
NCT03357757	Valproic acid, Avelumab	II	P16+ PSCC	Active, not recruiting
NCT03074513	Valproic acid, Avelumab	II	Refractory/metastatic PSCC	Active, not recruiting
NCT02379520	HPV-specific T-cells, Cytoxan, fludarabine, Nivolumab	I	Relapsed HPV-associated cancers	Active, not recruiting
NCT03439085	MEDI0457 (DNA-based cancer vaccine), Durvalumab	II	Recurrent/metastatic HPV- associated cancers	Active, not recruiting
NCT04432597	PRGN-2009 (HPV vaccine) ± M7824 (bifunctional anti-PD-L1/TGFβ Trap)	I/II	Locally advanced or metastatic HPV-associated malignancies	Active, not recruiting
NCT02308241	Ribavirin	NA	Recurrent/metastatic HPV- associated cancers	Completed
NCT03427411	M7824 (bifunctional anti-PD-L1/TGFβ Trap)	II	Locally advanced or metastatic HPV-associated malignancies	Completed
NCT03418480	HPV anti-CD40 RNA vaccine	I/II	Relapsed/refractory HPV-16+ penile carcinoma	Completed
NCT03912831	E7 TCR T-cells (KITE-439), cyclophosphamide, fludarabine, interleukin-2	I	(HLA)-A*02:01+ with relapsed/ refractory HPV16+ cancers	Terminated
NCT03618953	Ad-E6E7 (mutant form of HPV), MG1-E6E7 (oncolytic Maraba virus), Atezolizumab	I/Ib	Recurrent or metastatic HPV-associated tumors	Terminated

* National Clinical Trial number.

## Data Availability

No new data were created or analyzed in this study. Data sharing is not applicable to this article.
